# Recurrent gastric cancer sustaining a partial response after the nivolumab discontinuation because of immune-related adverse events: a case report

**DOI:** 10.1186/s40792-020-01050-1

**Published:** 2020-10-19

**Authors:** Takaaki Arigami, Daisuke Matsushita, Keishi Okubo, Takako Tanaka, Ken Sasaki, Masahiro Noda, Yoshiaki Kita, Shinichiro Mori, Yusuke Tsuruda, Hiroshi Kurahara, Takao Ohtsuka

**Affiliations:** 1 Department of Onco-Biological Surgery, Kagoshima University Graduate School of Medical and Dental Sciences, 8-35-1 Sakuragaoka, Kagoshima, 890-8520 Japan; 2 Department of Digestive Surgery, Breast and Thyroid Surgery, Kagoshima University Graduate School of Medical and Dental Sciences, Kagoshima, Japan

**Keywords:** Nivolumab, Immune-related adverse events, Tumor response, Gastric cancer

## Abstract

**Background:**

The prognosis of patients with recurrent gastric cancer is poor despite chemotherapy being clinically recommended as the first therapeutic strategy. Recent clinical trials have established the clinical utility of nivolumab in the third-line treatment of such patients. Immune-related adverse events (irAE) have been focused as a promising predictor for tumor response to nivolumab. This report aims to present a long-term survivor of recurrent gastric cancer who was followed up without any treatments after the nivolumab discontinuation because of irAE.

**Case presentation:**

A 65-year-old male with stage III gastric cancer (cT4aN1M0) underwent surgery after neoadjuvant chemotherapy. Owing to the final pathological stage IIIB (ypT4bN1M0), the patient received adjuvant chemotherapy. Nevertheless, during adjuvant chemotherapy 1-year post-surgery, computed tomography (CT) revealed lymph node swelling in station no. 9. Thus, upon diagnosis with lymph node recurrence, the patient was treated with two courses of capecitabine + oxaliplatin and three courses of ramucirumab + paclitaxel as the first- and second-line regimens, respectively. Based on these regimens, the patient had a progressive disease to chemotherapy. Consequently, we administered nivolumab as the third-line regimen. After four courses of nivolumab, CT revealed a significant shrinkage of the metastatic lymph node, with a 45.6% reduction. We confirmed a partial response during 11 courses of nivolumab. Since the occurrence of grade 4 diabetes mellitus after 12 courses of nivolumab, the patient was followed up without any treatment after the nivolumab discontinuation. Currently, the patient remains a partial response for 15 months, since the nivolumab discontinuation and is alive for 31 months after disease recurrence.

**Conclusions:**

Acute irAE during nivolumab chemotherapy could be one of the crucial clinical factors to predict tumor suppression in patients with advanced gastric cancer.

## Background

Gastric cancer is one of the leading gastrointestinal malignancies in Japan. The recent advancement of chemotherapy led to significant prognostic improvements in patients with unresectable advanced or recurrent gastric cancer [1]. Some studies focused on immunotherapy as a novel promising tool in patients with various malignancies, including gastric cancer [2–5]. Nivolumab, an anti-programmed cell death protein 1 antibody, is one of the representative immune checkpoint inhibitors. A randomized, double-blind, placebo-controlled, phase 3 study (ATTRACTION-2) on patients with gastric or gastroesophageal junction cancer previously treated with ≥ 2 chemotherapy regimens reported that the 2-year overall survival rates in the nivolumab and placebo groups were 10.6% and 3.2%, respectively [5]. Accordingly, the Japanese Gastric Cancer Treatment Guidelines 2018 stipulates that nivolumab is the third-line recommended regimen in patients with unresectable advanced or recurrent gastric cancer [6].

Although several studies have investigated useful biomarkers for predicting tumor response to nivolumab in patients with gastric cancer [7–9], no potential predictive marker has been identified to date. Besides, severe immune-related adverse events (irAE) are a crucial issue in the clinical management of patients with gastric cancer who undergo nivolumab chemotherapy [10]. A recent study reported a correlation between the presence or absence of irAE and the clinical benefit for nivolumab-treated patients with gastric cancer [11]. Here, we report a long-term survivor of recurrent gastric cancer maintaining a partial response (PR) after the nivolumab discontinuation because of irAE.

## Case presentation

A 65-year-old male was referred to our hospital and clinically diagnosed with stage III gastric cancer (cT4aN1M0). The patient was enrolled in the phase II clinical trial titled as perioperative chemotherapy with intraperitoneal (IP) and intravenous (IV) paclitaxel (PTX) plus S-1 for serosa positive gastric cancer (UMIN000013109). Therefore, the patient received three courses of IP and IV PTX + S-1; this regimen comprised a 3-week course of oral S-1 (80 mg/m^2^/day) on days 1–14, with IP PTX (20 mg/m^2^) and IV PTX (50 mg/m^2^) on days 1 and 8 [12]. Based on the Response Evaluation Criteria in Solid Tumors (RECIST) [13], the patient without target lesions had a non-progressive disease and non-complete response. Thus, the patient underwent distal gastrectomy with D2 lymphadenectomy. However, as the tumor directly invaded into the transverse colon, he underwent a partial resection of the transverse colon. The pathological examination confirmed stage IIIB gastric cancer (ypT4bN1M0). Postoperatively, the patient received three courses of IP and IV PTX + S-1, followed by adjuvant chemotherapy with oral S-1.

**Fig. 1:**
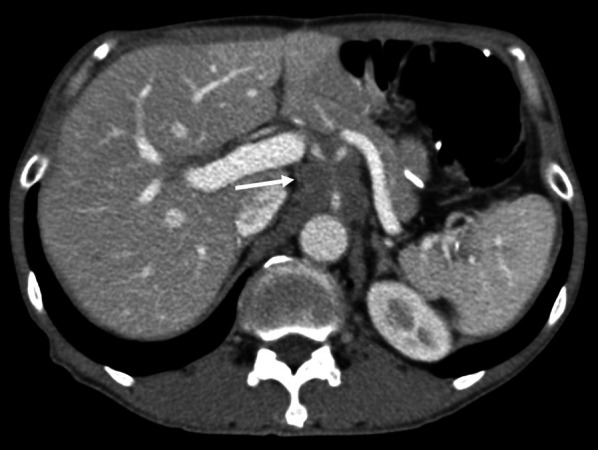
Computed tomography revealed a swollen lymph node (35 mm × 33 mm) in station no. 9 along the celiac artery (arrow)

**Fig. 2:**
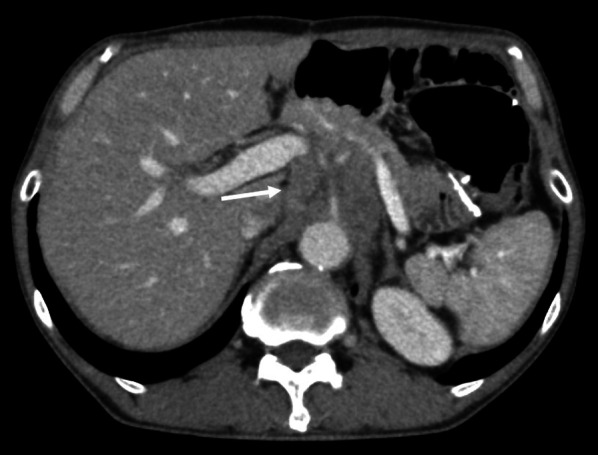
After two courses of capecitabine + oxaliplatin, the size of the metastatic lymph node was 47 mm × 43 mm (arrow)

**Fig. 3:**
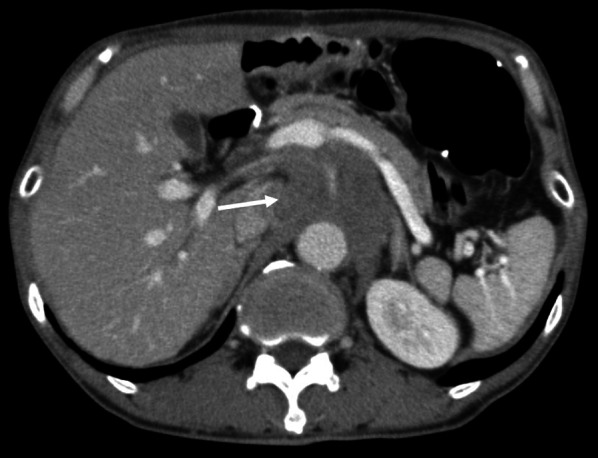
After three courses of ramucirumab + paclitaxel, the size of the metastatic lymph node was 58 mm × 57 mm (arrow)

**Fig. 4:**
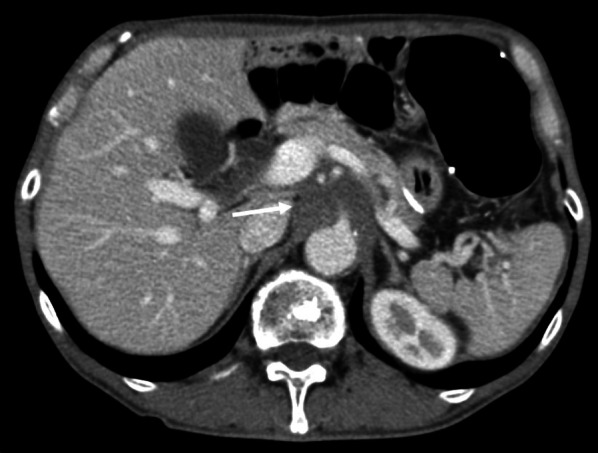
After four courses of nivolumab, the size of the metastatic lymph node was 34 mm × 31 mm (arrow)

While receiving adjuvant chemotherapy 1-year post-surgery, enhanced computed tomography (CT) revealed lymph node swelling in station no. 9 along the celiac artery (Fig. 1). Upon diagnosed with lymph node recurrence, the patient was treated with two courses of capecitabine + oxaliplatin without trastuzumab because of a human epidermal growth factor receptor 2 (HER2)-negative gastric cancer. The regimen comprised oral administration of capecitabine 1800 mg/day, two times daily for the first 2 weeks of the 3-week cycle and 130 mg/m^2^ of IV oxaliplatin on day 1 of each cycle. However, abdominal CT revealed further progression of lymph node metastasis, with a 30.3% increment (Fig. 2). As the second-line regimen, the patient received ramucirumab (RAM) + PTX, including a 28-day course of IV RAM (8 mg/kg) on days 1 and 15 + IV PTX (80 mg/m^2^) on days 1, 8, and 15. After three courses of RAM + PTX, enhanced CT revealed enlargement of lymph node metastasis, with a 32.6% increment (Fig. 3). Owing to the patient’s progressive disease in the tumor response to the second-line chemotherapy, nivolumab 3 mg/kg was administered every 2 weeks and 240 mg/body every 2 weeks after September 2018 as the third-line regimen. After four courses of nivolumab, CT revealed a significant shrinkage of the metastatic lymph node, with a 45.6% reduction (Fig. 4).

**Fig. 5:**
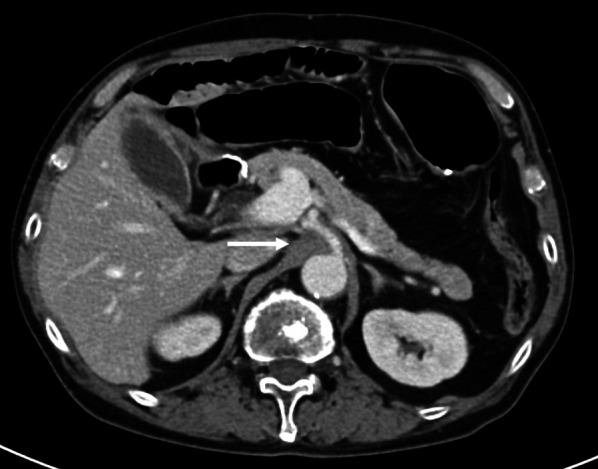
After 15 months since the nivolumab discontinuation, the size of the metastatic lymph node was 15 mm × 14 mm (arrow)

Consequently, the patient exhibited a partial response (PR) based on the RECIST. During 11 courses of nivolumab, enhanced CT confirmed a PR. However, after 12 courses of nivolumab, the patient was diagnosed with grade 4 diabetes mellitus, resulting in the nivolumab discontinuation because of severe irAE. Accordingly, insulin was administered for nivolumab-induced type 1 diabetes mellitus, which promptly regulated the plasma glucose level. Finally, the patient was followed up without any treatment after the nivolumab discontinuation. Abdominal CT after 15 months, since the nivolumab discontinuation revealed a PR (Fig. 5), and the patient controls tumor progression under treatment-free and is alive for 31 months since disease recurrence.

## Discussion

This report presents a case of recurrent gastric cancer sustaining tumor shrinkage despite the nivolumab discontinuation because of irAE. To the best of our knowledge, this is the first case report of recurrent gastric cancer exhibiting a partial clinical response to nivolumab for 15 months since its discontinuation.

Based on the 2018 Japanese Gastric Cancer Treatment Guidelines, the recommended chemotherapeutic regimens are defined by the HER2 expression status [6]. Our patient with HER2-negative recurrent gastric cancer received two courses of capecitabine + oxaliplatin without trastuzumab as the first-line chemotherapy, followed by three courses of RAM + PTX as the second-line chemotherapy. Regrettably, none of these chemotherapeutic regimens exhibited clinical utility in our case. Thus, our patient received nivolumab as the third-line chemotherapy. The 2-year update data of the ATTRACTION-2 trial reported that the objective response rates in the nivolumab and placebo groups were 11.9% (32/268) and 0% (0/131), respectively, while the disease control rates were 40.3% (108/268) and 25.2% (33/131), respectively [5]. Surprisingly, our patient already exhibited a PR in the initial assessment for nivolumab after RAM + PTX. Indeed, some recent studies have suggested the clinical benefit of nivolumab after RAM, an anti-vascular endothelial growth factor receptor 2 monoclonal antibody, in patients with advanced gastric cancer [14, 15]. Tada et al. revealed that RAM-containing therapy induced both programmed death-ligand 1 (PD-L1) expression and CD8^+^ T-cell infiltration [14]. These findings suggested that RAM could have clinical utility as an immunomodulator in combination with immune checkpoint inhibitors. Thus, nivolumab after RAM-containing therapy could be a promising tool in the strategic management of patients with unresectable advanced or recurrent gastric cancer.

Some recent studies have paid attention to the predictive markers for tumor response to nivolumab in patients with gastric cancer [7, 9, 11, 16]. In addition, several studies have proposed the neutrophil-to-lymphocyte ratio, Eastern Cooperative Oncology Group Performance Status, PD-L1 status, and mismatch repair as potential markers for predicting tumor response to nivolumab [7, 9, 16]. However, our patient had type 1 diabetes mellitus after 12 courses of nivolumab and has been followed up without any treatments since then. Our patient exhibited a PR, even in recent CT, 15 months after the nivolumab discontinuation. This finding suggests that recurrent tumors are controlled for a prolonged time without therapeutic interventions. Usually, this phenomenon is rare in chemotherapy using conventional cytotoxic agents. Osa et al. reported that nivolumab binding on memory T cells was identified more than 20 weeks after the final dose [17]. Further understanding of immunokinetics would allow the development of immunotherapy via immune checkpoint molecules. Lately, irAE has been garnering considerable attention as a promising predictor for identifying responders to nivolumab [11]. The ATTRACTION-2 trial suggested that the incidence of treatment-related adverse events of any grade and grade 3–4 in the nivolumab group was 43.0% and 11.8%, respectively, as well as the incidence of irAE, such as colitis, diabetes, hepatitis, hypophysitis, and hypothyroidism, is low [5]. Masuda et al. reported that the median overall survival in the irAE and non-irAE groups was 16.8 and 3.2 months, respectively (*P* < 0.001) [11]. Furthermore, the median progression-free survival (PFS) in the irAE and non-irAE groups was 7.5 and 1.4 months, respectively (*P* < 0.001) [11]. The multivariate analysis revealed that the presence or absence of irAE significantly correlated with prognosis [11]. Our patient is maintaining a PR with a PFS of 15 months. All the findings mentioned above strongly establish a close correlation between the presence or absence of irAE and tumor response in patients with unresectable advanced or recurrent gastric cancer receiving nivolumab.

## Conclusions

In conclusion, this report suggests that the presence or absence of irAE during nivolumab chemotherapy could correlate with tumor response and prognosis in patients with gastric cancer.

## Data Availability

The datasets obtained during this study are available from the corresponding author on reasonable request.

## Abbreviations

CT: Computed tomography
HER2: Human epidermal growth factor receptor 2
IP: Intraperitoneal
irAE: Immune-related adverse events
IV: Intravenous
PD-L1: Programmed death-ligand 1
PR: Partial response
PTX: Paclitaxel
RAM: Ramucirumab
RECIST: Response Evaluation Criteria in Solid Tumors
